# Common capacity-limited neural mechanisms of selective attention and spatial working memory encoding

**DOI:** 10.1111/j.1460-9568.2011.07794.x

**Published:** 2011-09

**Authors:** Fabian Fusser, David E J Linden, Benjamin Rahm, Harald Hampel, Corinna Haenschel, Jutta S Mayer

**Affiliations:** 1 Department of Psychiatry, Psychosomatic Medicine, and Psychotherapy, J.W. Goethe-University60528 Frankfurt, Germany; 2 School of Psychology, Cardiff UniversityTower Building, 70 Park Place, Cardiff CF10 3AT, UK; 3 Institute of Medical Psychology, J.W. Goethe-University60528 Frankfurt, Germany; 4 Medical Psychology and Sociology, University Medical Center, Gutenberg University55128 Mainz, Germany; 5 Department of Psychology, City UniversityLondon EC1V0HB, UK; 6 Department of Psychology, Vanderbilt UniversityNashville, TN 37240, USA

**Keywords:** attention, cognitive processing, eye movements, fMRI, working memory

## Abstract

One characteristic feature of visual working memory (WM) is its limited capacity, and selective attention has been implicated as limiting factor. A possible reason why attention constrains the number of items that can be encoded into WM is that the two processes share limited neural resources. Functional magnetic resonance imaging (fMRI) studies have indeed demonstrated commonalities between the neural substrates of WM and attention. Here we investigated whether such overlapping activations reflect interacting neural mechanisms that could result in capacity limitations. To independently manipulate the demands on attention and WM encoding within one single task, we combined visual search and delayed discrimination of spatial locations. Participants were presented with a search array and performed easy or difficult visual search in order to encode one, three or five positions of target items into WM. Our fMRI data revealed colocalised activation for attention-demanding visual search and WM encoding in distributed posterior and frontal regions. However, further analysis yielded two patterns of results. Activity in prefrontal regions increased additively with increased demands on WM and attention, indicating regional overlap without functional interaction. Conversely, the WM load-dependent activation in visual, parietal and premotor regions was severely reduced during high attentional demand. We interpret this interaction as indicating the sites of shared capacity-limited neural resources. Our findings point to differential contributions of prefrontal and posterior regions to the common neural mechanisms that support spatial WM encoding and attention, providing new imaging evidence for attention-based models of WM encoding.

## Introduction

Visual working memory (WM), the ability to retain information for short periods of time thus making it available for manipulation, is essential in the active guidance of behaviour (Baddeley, 1986). One characteristic feature of WM is its severe capacity limitation. Humans are able to actively maintain up to only four items (Cowan, 2001), an estimate that is highly similar to the capacity estimate for visual selective attention (Pylyshyn & Storm, 1988; Scholl, 2001). Accordingly, attention-based models of WM regard selective attention as the capacity-limited process that constrains the capacity of visual WM (Cowan, 2001; Rensink, 2002; Wheeler & Treisman, 2002). Functional imaging studies have revealed considerable overlap between the neural substrates for visual WM and attention, for example in the frontal and parietal lobes (Culham *et al.*, 2001; Corbetta & Shulman, 2002; Wager & Smith, 2003; Pessoa & Ungerleider, 2004). Specifically, the neural substrates of the capacity constraints of visual WM have been localized in the posterior parietal cortex (PPC; Linden *et al.*, 2003; Todd & Marois, 2004). Xu & Chun (2006) further dissociated the roles of the superior and inferior intraparietal sulcus (IPS) for visual WM capacity, arguing that the latter might subserve a spatial attention mechanism that selects and determines the maximum number of objects held in visual WM. Furthermore, there is evidence that, under certain perceptual conditions, activity in the IPS has a capacity limit similar to that of visual object-based WM (Mitchell & Cusack, 2008). Taken together, these findings raise the possibility that activity in PPC seen in WM tasks reflects attention-related processes, hence supporting an attention-based model of visual WM. Previous neuroimaging studies investigating the role of posterior brain regions in WM capacity have largely focused on the nonspatial component of visual WM (Linden *et al.*, 2003; Mayer *et al.*, 2007a). However, at the behavioural level there is strong evidence for interference between spatial attention and spatial WM (Smyth & Scholey, 1994; Awh & Jonides, 2001; Oh & Kim, 2004; Woodman & Luck, 2004).

The overlap of brain activation to attention and WM load (LaBar *et al.*, 1999; Pollman & von Cramon, 2000; Corbetta *et al.*, 2002; Lepsien *et al.*, 2005; Ikkai & Curtis, 2011; Soto *et al.*, 2008) alone does not provide sufficient evidence for shared or interacting processes. Such overlap can be a result of additive activation increases to the different manipulations of interest.

The present experiment was therefore motivated by the need to manipulate the demand on WM and attention within one single task and to identify brain regions which would show an interaction effect. Such an interaction effect would provide strong evidence for common cognitive and neural resources shared by spatial WM encoding and spatial attention. Participants were presented with a search array and performed easy or difficult visual search (ES and DS, respectively) in order to encode one, three or five locations into WM (WM load 1, 3, 5). Assuming that the blood oxygen level-dependent (BOLD) signal is a linear function of the number of items held in WM and attentional load, at least within certain boundaries, we made the following predictions with regard to common limited neural resources. If spatial WM and attention shared common capacity-limited neural resources, these resources would become exhausted in conditions that make high demand on both processes (DS/WM load 5). In that case, we expected to find an interaction effect between attentional demand and WM load, i.e. a less than additive increase in BOLD activation with increasing demands on WM and visual search. Conversely, regions that mediated both processes and were well within their processing limits would be associated with main effects for both task manipulations and an additive increase in BOLD activation under simultaneous WM and attentional demands.

## Materials and methods

### Participants

Thirty-one healthy participants (17 females, mean age 27.6 ± 4.0, range 20–35 years) were recruited from an academic environment and volunteered in this study. Participants reported normal or corrected-to-normal visual acuity, normal color vision and no history of neurological or psychiatric illness. The study was approved by the local ethics committee. All participants gave written informed consent.

### Stimuli, task and procedure

**Fig. 1:**
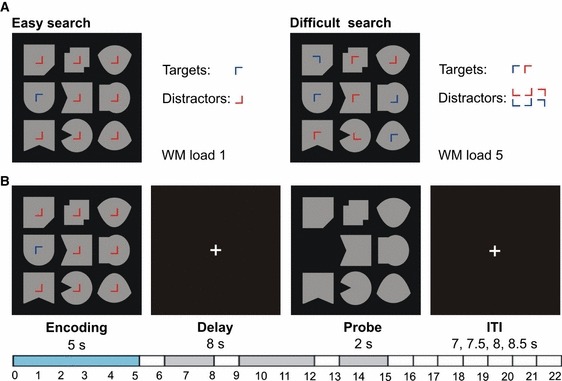
(A) Stimuli and (B) trial design. Participants were presented with a search array for 5 s and asked to memorize only the positions marked with a target item. The targets were either easy to discriminate from the distractors (ES) or not (DS). WM load was manipulated by changing the number of targets (load 1, left array; load 5, right array; load 3 not shown). The analysis focused on the encoding predictor.

We used the same stimuli as in our previous studies investigating interactions between attention and object WM encoding on behavioural (Mayer *et al.*, 2007b) and neural (Mayer *et al.*, 2007a) levels. In the current experiment our task combined visual search and delayed discrimination of locations. The search array consisted of nine different task-irrelevant grey geometric shapes (each spanning approximately 2.4° × 2.4° of visual angle), arranged in a 3 × 3 matrix, and presented in the center of the screen and on a black background (Fig. 1A). In the center of each shape we placed a small L-shaped item (0.65° × 0.65°) which could appear in one of four different orientations (rotated by 0°, 90°, 180° or 270°, clockwise) and was colored either blue or red. Participants needed to memorize only the locations associated with an L oriented at 90° (target items). The locations associated with Ls of other orientations could be ignored (distractor items). The shapes surrounding the target and distractor items were task-irrelevant and were only included to be able to compare the results with our previous findings on object WM encoding. To manipulate the demand for attentional selection we implemented two search conditions in which target items had either unique features (ES; low attentional demand) or shared most of their features with the distractors (DS; high attentional demand; Treisman & Gormican, 1988; Duncan & Humphreys, 1989). In the ES condition target Ls always appeared in blue and distractors in red. Distractor Ls were always oriented at 270°. In contrast, in the DS condition each target and distractor was assigned randomly either blue or red color. Also, each distractor’s orientation was selected randomly from the three nontarget orientations (0°, 180° and 270°). Increasing the similarity between targets and distractors (Treisman & Gormican, 1988; Duncan & Humphreys, 1989) results in considerably longer search times, reflecting increasing demands on selective attention in the DS condition (Mayer *et al.*, 2007b). The search array contained one, three or five targets. Only the locations of these targets needed to be encoded (WM loads 1, 3 and 5).

Each trial began with the presentation of the search array for 5 s (Fig. 1B). After an 8-s delay interval showing a fixation cross the original stimulus array was presented for 2 s without the center items and with one of the background shapes missing. Participants responded with a left- or right-hand button press to indicate whether the location of the missing shape did or did not match one of the target locations. Thus, only WM for target locations but not the identity of the missing shape was probed. Half of the trials were matches. Presenting the search array for a fixed amount of time was crucial to rule out differences in brain activation being explained by differences in sensory stimulation. Moreover, as our primary goal was to identify shared capacity-limited neural resources for visual search and spatial WM encoding we chose a rather long encoding period of 5 s, which would allow participants to engage successfully in the process of WM encoding even in the DS condition. This duration was based on a previous study in which we directly assessed the time that was needed to encode the target locations into WM while engaging ES or DS (Mayer *et al.*, 2007b). The intertrial interval (ITI) again presenting a fixation cross was jittered (lasting 7, 7.5, 8 or 8.5 s) to minimise multicollinearity, which is successful even with shorter ITIs (Cairo *et al.*, 2004), using steps of half the TR in order to increase the effective sampling rate, resulting in total trial durations between 22 and 23.5 s. The experiment consisted of four runs with 30 experimental trials each, resulting in 20 repetitions for each of the six trial types (load 1/ES; load 3/ES; load 5/ES; load 1/DS; load 3/DS; load 5/DS). ES and DS conditions were presented in separate blocks of seven or eight trials (two blocks for each condition per run) in a pseudorandomized order across runs. Before starting a new block, participants were given an instruction about the targets they needed to search for. Participants were instructed to fixate during the experiment. However, the majority of subjects reported that keeping fixation was difficult while searching for the targets. Within each block, WM load conditions were presented in a pseudorandomised order to equal the number of WM load 1, 3 and 5 trials. Twenty per cent of the trials (four trials of each condition) were partial trials that ended after the encoding phase without informing participants in advance. In this case, the word ‘relax’ was presented following the presentation of the array. Thus, in these trials participants were required to encode the target locations into WM but not to maintain them during the delay, and to retrieve them during the probe phase. These trials were pseudorandomly interspersed and were included to compensate for the overlap of the hemodynamic responses to successive neural events associated with the encoding and maintenance phases (Ollinger *et al.*, 2001). Instructions were given outside the scanner. Prior to scanning, participants performed two practice blocks of 10 trials, one for each of the two search conditions.

WM capacity (*K*) was estimated for each load condition using Cowan’s formula: *K* = (hit rate + correct rejection rate−1) × *N*, where *N* is the number of targets presented (Cowan, 2001). This approach allows quantification of the number of items held in memory, *K*, from a set size of *N* items. Therefore, this measure is typically interpreted as *K* items being encoded with high fidelity, with no encoding of any other items.

### Image acquisition and analysis

Anatomical three-dimensional T1-weighted images (voxel size 1.0 × 1.0 × 1.0 mm^3^) and functional images were acquired on a 3-T Magnetom Trio scanner (Siemens Medical Systems, Erlangen, Germany) equipped with a standard head coil. Functional images were collected using 17 axial slices (5 mm thickness with 3.6 × 3.6 mm in-plane resolution, gap 0.5 mm) covering the whole brain with a BOLD-sensitive EPI sequence: repetition time (TR), 1 s; echo time (TE), 30 ms; flip angle (FA), 80°, field of view (FOV), 230 mm; matrix size = 64 × 64; duration of each run, 667 s. Trials were triggered by scanner pulses and presented with the Experimental Run-Time System software (ERTS; Berisoft, Frankfurt, Germany). Stimuli were back-projected from an LCD projector onto a screen viewed through a mirror by the supine subject in the MR scanner.

Image analyses were performed with Brainvoyager QX, version 2.1.2 (Brain Innovation, Maastricht, The Netherlands). Data preprocessing included slice scan time correction with sinc interpolation, 3-D motion correction, spatial smoothing with a 4-mm Gaussian kernel (full width at half-maximum), temporal high-pass filtering with a cutoff of 222 s, and linear trend removal. The functional and structural 3-D data sets were transformed into Talairach space. The general linear model was computed for 119 normalised volume time courses based on a percentage signal change transformation approach. The data from five runs of three participants were excluded from the analysis due to technical problems during the scanning procedure. For the design matrix, four time points were defined per experimental condition, representing the different periods of each experimental trial (encoding, 0–5 s after stimulus onset; early delay, 6–8 s; late delay, 9–12 s; retrieval, 13–15 s; Fig. 1B). The early delay predictor was included to ensure that the activity captured by the late delay predictor was not contaminated by encoding activity (Zarahn *et al.*, 1997) and therefore was not further analysed. Predictors were convolved with a gamma function model of the hemodynamic response peaking after 5 s (Friston *et al.*, 1998). All error trials were collapsed on a separate predictor.

The resulting parameter maps from each subject were entered into a second-level whole-brain repeated-measurements anova with subjects as a random factor and the within-subject factors of attentional demand (level 1, ES; level 2, DS) and WM load (level 1, load 1; level 2, load 3; level 3, load 5). Main effects of attentional demand and WM load and the interaction effect between the two factors were tested based on *F*-statistics. Analyses were performed only on voxels showing an increase in the mean activity across conditions from baseline during search using a mask that contrasted BOLD activity during the encoding phase against baseline activity (load 1/ES encoding + load 3/ES encoding + load 5/ES encoding + load 1/DS encoding + load 3/DS encoding + load 5/DS). Statistical maps were thresholded at *q* < 0.05, corrected for false discovery rate (Genovese *et al.*, 2002), and visualised on a surface reconstruction of the MNI template brain (courtesy of the Montreal Neurological Institute). Averaged event-related fMRI time courses are shown for selected regions of interest (ROIs) where the effects of WM load and attentional demand appeared most prominently. ROIs were functionally defined based on the multisubject statistical volume maps. Peak activation defined the centers of ROIs that comprised a 5 × 5 × 5 mm³ cuboid each. Representative time courses for each experimental condition were obtained by averaging the percentage signal changes of the individual voxels within the obtained volume across all participants and repetitions.

### Eye movement recordings and analysis

The primary aim of the present study was to investigate the role of frontal and occipitoparietal regions for the neural resources shared by spatial attention and the encoding of information into spatial WM. A further aim of this study was to differentiate between attention-related and saccade-related brain activations. Saccadic eye movements have been associated with activation in the precentral sulcus (PrcS) at the junction with the superior frontal sulcus (SFS), corresponding to the frontal eye fields (FEF; Paus, 1996; Goebel *et al.*, 1998) and the PPC (Sereno *et al.*, 2001; Schluppeck *et al.*, 2005). These regions largely overlap with those reported in tasks of spatial attention (Corbetta *et al.*, 1998; Nobre *et al.*, 2000; Perry & Zeki, 2000; Beauchamp *et al.*, 2001; Grosbras *et al.*, 2005; Ikkai & Curtis, 2008; Fairhall *et al.*, 2009). Furthermore, the number of eye movements increases from low to high attention-demanding visual search tasks (Zelinsky & Sheinberg, 1997; Maioli *et al.*, 2001). Therefore, we expected significantly more eye movements during DS trials than during ES trials and as a consequence a considerable degree of overlap in activation related to visual attention and the execution of eye movements.

Eye movements were monitored in 10 of our 31 subjects using an infrared-based ASL 504 eye-tracking system (Applied Science Laboratories, Waltham, MA, USA) with a long-range optics module adapted to the MR environment. Eye data from two subjects had to be discarded due to insufficient data quality.

In each measurement, the eye position of the right eye was recorded at a sampling rate of 60 Hz and with a spatial resolution of approximatly 0.25° visual angle and an accuracy of 0.5° visual angle. Calibration was performed prior to each session and repeated between functional runs if necessary. Eye movement recordings were triggered by stimulus presentation.

For each trial we visualised and analysed eye data of the encoding phase (0–5 s after stimulus onset) using ILAB 3.6.4 (Gitelman, 2002). The analysis only included trials with > 70% valid data points; others were regarded as error trials due to blinks or other artifacts (104 of 899 correct trials; 11.5%). On average, the remaining trials contained 87.7% valid data points.

Saccades were detected automatically with an algorithm based on an initial velocity threshold of 30°/s, a saccade peak cutoff of 15% and a minimum duration of 30 ms. Additionally, visual inspection of each saccade was done to validate saccade onset and offset and to correct for possible errors of the algorithm. Only saccades with an amplitude > 1° visual angle were chosen to determine the number of saccades performed in a trial. The number of saccades for trials with missing data points (see above) was corrected by a mean imputation of saccades of the respective trial. The mean saccade frequency (number of saccades per trial) was then calculated for each of the six conditions.

To reveal neural activation attributable to eye movements, we computed a fixed-effects general linear model for 32 *z*-normalized volume time courses of eight participants. In comparison to the previous analysis, the design matrix contained one additional predictor modelling the number of saccades during the encoding phase (0–5 s after stimulus onset). For each trial the respective number of saccades was divided by the mean saccade frequency across all trials, and the ideal box-car response of the encoding period was then weighted with this relative value and convolved with the hemodynamic response function. The resulting saccade predictor accounts for activations which are linearly related to the number of saccades made. As it reflects the number of saccades independent of trial type, it correlated only very mildly with our task regressors (average *r* = 0.12). To compare activations between experimental conditions, linear contrasts were performed using *t*-statistics [attentional demand: (load 1/DS + load 3/DS + load 5/DS)−(load 1/ES + load 3/ES + load 5/ES); WM load: (load 5/ES + load 5/DS)−(load 1/ES + load 1/DS); interaction effect: (load 5/ES−load 1/ES)−(load 5/DS−load/1DS)]. Multi-subject statistical maps of the analysis were thresholded at *q* < 0.05, corrected for false discovery rate and visualised on a flatmap of the MNI template brain.

## Results

### Behavioural performance at test

**Fig. 2:**
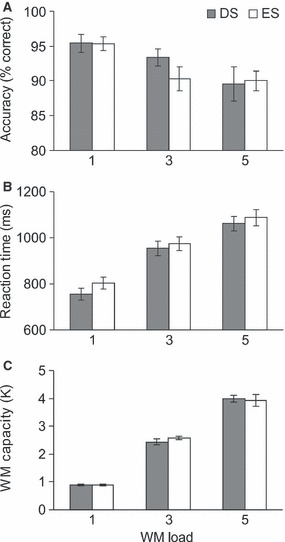
Behavioural results. (A) Mean response accuracy, (B) reaction time, and (C) WM capacity (*K*) in the six experimental conditions. Bars represent SEM.

Participants’ WM performance at test was equally good under ES and DS (WM load 1, 95.4 and 95.4% correct, respectively; WM load 3, 90.3 and 93.3% correct; WM load 5, 90.0 and 89.6%; anova, *F*_1,30_ = 1.01, *P* = 0.32; Fig. 2A). Similarly, WM capacity (*K*) did not differ between ES and DS conditions (*F*_1,30_ = 0.25, *P* = 0.62; Fig. 2C). There was a main effect of the factor search difficulty on RTs (*F*_1,30_ = 16.97, *P* < 0.001; Fig. 2B). However, post hoc *t*-tests revealed a significant difference between the ES and DS conditions only within WM load 1 (WM load 1, 804 and 756 ms, respectively; *t*_30_ = 3.78, *P* < 0.01; WM load 3, 972 and 955 ms; *t*_30_ = 1.50, *P =* 0.14; WM load 5, 1087 and 1062 ms; *t*_30_ = 1.65, *P =* 0.11).

A strong main effect was observed for WM load. In both search conditions response accuracy declined from WM load 1 to WM load 5 (on average by 5.6 percentage points; *F*_2,60_ = 8.34, *P* < 0.05), and RTs were significantly slower (on average by 289 ms; *F*_2,60_ = 158.29, *P* < 0.001). *Post hoc t*-tests of differences between successive levels of WM load indicated that accuracy was significant lower for WM load 5/ES vs. load 1/ES (*t*_30_ = 3.57, *P* < 0.01), load 3/ES vs. load 1/ES (*t*_30_ = 2.61, *P* < 0.05) and load 5/DS vs. load 1/DS (*t*_30_ = 2.34, *P* < 0.05; all other *t*-values < 1.65, *P*-values > 0.11). In both search conditions, RTs were significantly slower for WM load 5 vs. load 1, for WM load 3 vs. load 1 and for WM load 5 vs. load 3 (all *t*-values > 6.12, all *P*-values < 0.001). There was no significant interaction between search difficulty and WM load (*F*_2,60_ = 1.06, *P =* 0.35 for accuracy; *F*_2,60_ = 1.30, *P =* 0.28 for RTs). The findings that memory performance at test and WM capacity estimates did not differ between ES and DS conditions indicates that, due to the long encoding period, participants successfully engaged in the process of WM encoding even in the most demanding condition (WM load 5/DS). This was considered a prerequisite for probing activations for visual search and WM encoding.

### Brain systems for attention and encoding into spatial WM

**Fig. 3:**
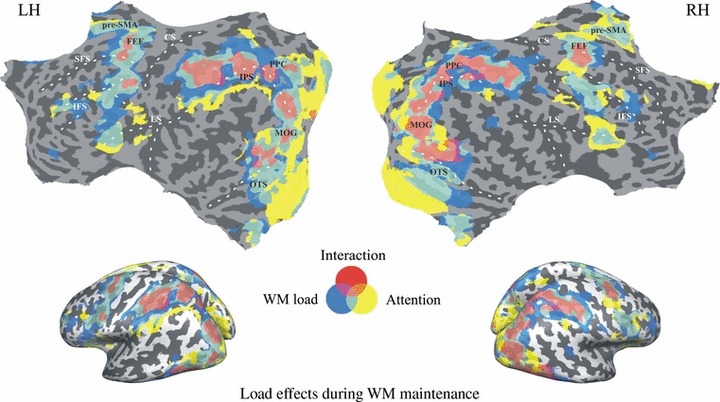
Group results (*n* = 31) for the encoding predictor (0–5 s). Statistical maps of the main effects for attentional demand (yellow), WM load (blue), and the significant two-way interaction of attentional demand × WM load (red) are projected on the flattened and inflated surface reconstruction of the MNI template brain (courtesy of the Montreal Neurological Institute) LH, left hemisphere; RH, right hemisphere. Activations are those exceeding a whole-brain false discovery rate threshold of *q*(FDR) < 0.05. CS, central sulcus; FEF, frontal eye field; IFS, inferior frontal sulcus; IPS, intraparietal sulcus; LS, lateral sulcus; MOG, middle occipital gyrus; OTS, occipitotemporal sulcus; PPC, posterior parietal cortex; pre-SMA, pre-supplementary motor area; SFS, superior frontal sulcus. For interpretation of color references in figure legend, please refer to the Web version of this article.

**Fig. 4:**
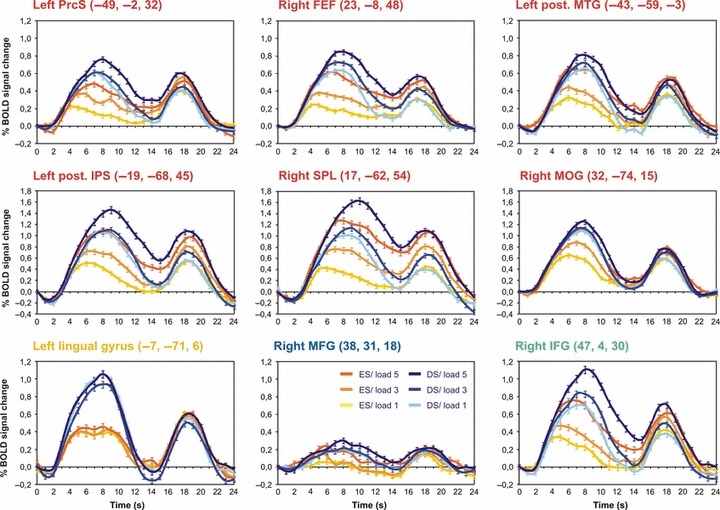
Averaged event-related time courses of the BOLD signal in the six experimental conditions from selected regions showing an interaction effect (red), an effect of attentional demand (yellow), an effect of WM load (blue), and effects of both manipulations (green). Bars represent SEM. FEF, frontal eye field; IFG, inferior frontal gyrus; IPS, intraparietal sulcus; MFG, middle frontal gyrus; MOG, middle occipital gyrus; MTG, middle temporal gyrus; post., posterior; PrcS, precentral sulcus; SPL, superior parietal lobule. For interpretation of color references in figure legend, please refer to the Web version of this article.

The analyses of fMRI data for the encoding predictor (0–5 s after stimulus onset) revealed a high degree of overlap in the brain areas that showed a significant main effect of visual search difficulty and those that showed a significant main effect of WM load. Overlap in activation with higher activation for DS vs. ES and higher activation with increasing WM load was observed bilaterally in the lateral occipitotemporal cortex, medial occipital cortex and lateral and medial parts of the parietal cortex (Figs 3 and 4, green color; Table 1). Overlapping frontal activation occurred along the PrcS extending into parts of the PFC, in the frontal midline and in the anterior insula. Subcortical activations were found in the thalamus, the basal ganglia and the superior colliculus.

**Table 1:** Brain regions showing significant activation for main and interaction effects during encoding

		Talairach coordinates (mm)			Effect *F*-values		
			
Brain region	BA	*x*	*y*	*z*	WM load	Attentional demand	Two-way interaction
Common activation							
L SFS	6	−22	−5	54	47.59	81.02	18.70
L dlPrcS	6	−31	−8	57	25.53	37.37	16.47
L FEF	6	−28	−11	45	24.96	40.62	14.56
R FEF	6	23	−8	48	57.67	67.57	31.18
L vlPrcS	6	−49	−2	32	48.70	82.54	15.99
R IFG	9	47	4	30	38.98	86.65	ns
L PrcG	6	−48	−7	39	22.75	33.22	ns
L SMA	6	−6	−2	53	32.86	31.96	ns
L pre-SMA	6	−3	8	49	26.95	18.86	ns
R pre-SMA	6	3	5	49	12.06	46.35	ns
L Insula	13	−34	13	9	30.21	96.02	ns
R Insula	13	30	18	9	19.84	99.75	ns
L SPL	7	−22	−59	51	44.36	54.24	37.21
R SPL	7	17	−62	54	53.17	27.94	44.03
L post. IPS	7	−19	−68	45	26.19	13.45	36.56
R post. IPS	7	26	−65	36	39.92	7.08	23.59
L IPL	40	−34	−41	39	38.54	27.94	19.74
L post. MTG	37	−43	−59	−3	19.98	26.35	17.89
R post. MTG	37	47	−65	3	14.58	36.98	17.19
L MOG	19	−28	−80	18	29.93	59.79	24.47
R MOG	19	32	−74	15	22.35	43.87	25.11
R Cuneus	17	11	−89	9	7.34	16.43	11.42
L Thalamus		−11	−15	9	23.48	86.20	ns
R Thalamus		8	−15	11	17.92	98.38	ns
L Globus pallidus		−13	3	6	49.49	40.85	ns
R Caudate nucleus		11	5	6	43.58	61.98	ns
L SC		−7	−26	−2	29.25	109.78	ns
R SC		6	−23	−3	36.54	114.69	ns
WM							
L SFG	6	−17	1	60	18.99	ns	ns
L MFG	9	−43	25	30	13.88	ns	ns
R MFG	46	38	31	18	12.44	ns	ns
L IFG	44	−46	4	15	29.80	ns	ns
L IPL	40	−45	−41	45	31.16	ns	ns
R IPL	40	42	−39	44	37.93	ns	ns
L Precuneus/SPL	7	−10	−58	59	27.56	ns	ns
R Precuneus/SPL	7	10	−56	58	21.23	ns	ns
R ITG	37	51	−54	−7	22.56	ns	ns
Attention							
L IFG	9	−34	10	23	ns	11.04	ns
R IFG	9	37	10	24	ns	11.91	ns
L Lingual gyrus	18	−7	−71	6	ns	106.90	ns
L Cuneus	18	−3	−79	7	ns	75.65	ns
R Cuneus	17	11	−68	12	ns	114.67	ns

Significant main and interaction effects (whole brain random-effects analysis) for the encoding predictor (0–5 s), *q*(FDR) < 0.05. Talairach coordinates of the activation maxima and respective *F*-values (WM load, attentional demand, and two-way interaction: WM load × attentional demand) are shown. BA, Brodmann area; dlPrcS, dorsolateral precentral sulcus; FEF, frontal eye field; IFG, inferior frontal gyrus; IPL, inferior parietal lobule; IPS, intraparietal sulcus; ITG, inferior temporal gyrus; MFG, middle frontal gyrus; MOG, middle occipital gyrus; MTG, middle temporal gyrus; PrcG, precentral gyrus; PrcS, precentral sulcus; pre-SMA, pre-supplementary motor area; SC, superior colliculus; SFG, superior frontal gyrus; SFS, superior frontal sulcus; SMA, supplementary motor area; SPL, superior parietal lobule; vlPrcS, ventrolateral precentral sulcus; ns, not significant.

Areas significantly responding to variations of attentional demand only were found most prominently in the lateral and medial occipital cortex (Figs 3 and 4, yellow; Table 1). Anterior parts of the lateral PFC bilaterally displayed significantly enhanced activation to increased WM load only (Figs 3 and 4, blue; Table 1). Please note that this does not imply significant functional selectivity of these activations for attentional processing as compared to WM and *vice versa*, but only that one effect attained significance whereas the other did not. The identification of functional selectivity of these activations was not the focus of this study and in consequence was not assessed.

### Interaction between WM load and attentional demand during encoding

Activation associated with a significant interaction effect between the factors attentional demand (DS, ES) and WM load (loads 1, 3 and 5) was found in a subset of the regions with overlapping activations for both effects. These bilateral regions included the lateral and medial parietal cortex along the IPS and the precuneus, and regions along the left ventral and bilateral dorsal PrcS including the FEF (Fig. 3, red color; Table 1). These regions showed a smaller increase in the BOLD signal with increasing WM load for DS as compared to ES (Fig. 4, red; Supporting Information Fig. S1). Thus, in both search conditions the BOLD response increased from WM load 1 to WM load 3. Activation further increased when participants needed to memorize five locations; however, this increase was smaller in the DS condition than the in the ES. The interaction appeared even more pronounced in visual cortex where the BOLD response increased from WM load 1 to load 3 and from load 3 to load 5 in the ES but were high and did not considerably differ across WM loads in the DS condition (Fig. 4; Supporting Information Fig. S1). Thus, in these regions the BOLD response did not exceed a plateau of activation that was reached with load 5/ES or with load 1/DS.

In contrast, in brain regions that showed an overlap in activation but no significant interaction (insula, frontal midline, lateral PFC, ventral PrcS and subcortical regions; Fig. 3, green color; Table 1), the increase in activation across WM load conditions did not differ between DS and ES conditions. Here, the BOLD signal additively increased with demands on WM load (load 1 vs. 3 vs. 5) and visual search (ES vs. DS; Fig. 4, green; Supporting Information Fig. S2).

### Load effects during WM maintenance

The primary goal of this study was to investigate interactions between attention-demanding visual search and spatial WM encoding. If participants successfully performed our WM task despite the concurrent demands on attentional resources, we expected to find an interaction between search difficulty and WM load during the encoding phase but not during the subsequent delay phase. Consistent with this hypothesis, the interaction contrast between search difficulty and WM load did not yield significant activation during the late delay phase (9–12 s after stimulus onset), even at a very lenient threshold of *P* < 0.01 (uncorrected), nor did delay activity increase in the DS condition as compared to the ES condition. Significantly stronger activation with increasing WM load was found in bilateral frontal and parietal regions. These activation foci were identical to those observed during the encoding phase, which revealed additional activation in prefrontal and ventral frontal regions and in early and higher visual areas.

### Oculomotor results

To rule out the possibility that the expected effects of increased attentional demand in the FEF and posterior parietal regions were primarily due to an increase in the number of saccades we recorded eye movements during the fMRI experiment and estimated differences in oculomotor activity between task conditions, in particular the ES and DS search conditions.

**Fig. 5:**
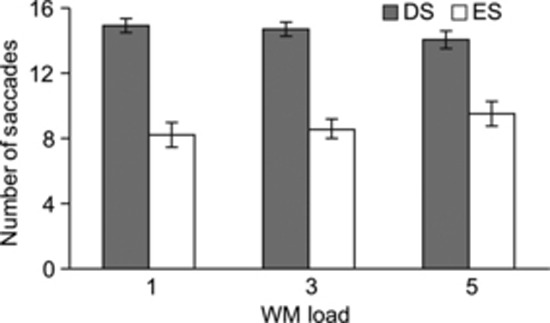
Mean number of saccades in the six experimental conditions. Bars represent SEM.

As expected saccade frequency was significantly higher in the DS vs. ES conditions [on average DS = 14.56 ± 0.27 (mean ± SEM), ES = 8.77 ± 0.41, anova, *F*_1,7_ = 171.68, *P* < 0.001; Fig. 5]. *Post hoc* comparisons found that the differences between DS and ES conditions were significant within each WM load condition (load 1/DS vs. load 1/ES, *t*_31_ = 11.40, *P* < 0.001; load 3/DS vs. load 3/ES, *t*_31_ = 12.36, *P* < 0.001; load 5/DS vs. load 5/ES, *t*_31_ = 8.25, *P* < 0.001). Overall, the number of saccades did not differ between WM load conditions (*F*_2,14_ = 0.17, *P =* 0.82). For ES, saccade frequency slightly increased with WM load (mean ± SEM: load 1/ES = 8.21 ± 0.77; load 3/ES = 8.59 ± 0.62; load 5/ES = 9.50 ± 0.74), whereas for DS the number of saccades slightly decreased with increasing WM load (mean ± SEM: load 1/DS = 14.95 ± 0.41; load 3/DS = 14.72 ± 0.43; load 5/DS = 14.02 ± 0.53) leading to a small but significant interaction between search difficulty and WM load (*F*_2,14_ = 8.55, *P* = 0.001).

### Brain systems for saccades

**Fig. 6:**
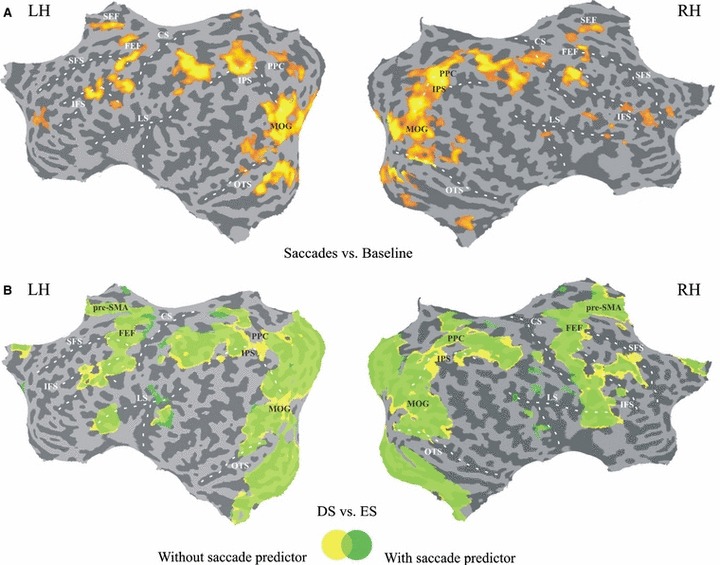
Group results (*n* = 8, GLM with additional saccade predictor; see Materials and Methods) for the encoding predictor (0–5 s). (A) Significant effect for the number of saccades (saccade predictor vs. baseline). (B) Statistical maps of the contrasts DS vs. ES with (green) and without (yellow) the additional saccade predictor. Activations are those exceeding a whole-brain false discovery rate threshold of *q*(FDR) < 0.05. CS, central sulcus; DS, difficult search; ES, easy search; FEF, frontal eye field; GLM, general linear model; IFS, inferior frontal sulcus; IPS, intraparietal sulcus; LS, lateral sulcus; MOG, middle occipital gyrus; OTS, occipito-temporal sulcus; PPC, posterior parietal cortex; pre-SMA, pre-supplementary motor area; SEF, supplementary eye fields; SFS, superior frontal sulcus. For interpretation of color references in figure legend, please refer to the Web version of this article.

fMRI analysis of the data from the eight subjects who underwent eye tracking indicated a significant effect for the number of saccades during the encoding phase in distributed frontal, occipitoparietal, occipitotemporal and subcortical regions (Fig. 6A, Table 2). As expected, saccade-related regions were highly similar to the areas reflecting a main effect of attentional demand (Fig. 6B, yellow). However, taking the saccade-related activation into account, the fixed-effects analysis of the data from the eight subjects revealed a highly similar effect of attentional demand both as compared to the fixed-effects analysis that did not include the saccade predictor and as compared to the random-effects analysis of the data from all participants [Fig. 6B (*n* = 8), with (green) and without (yellow) the saccade predictor, and Fig. 3 (*n* = 31), yellow]. Moreover, this was also the case for the effects of WM load and the interaction between the two factors [Supporting Information Fig. S3, (*n* = 8); Fig. 3 (*n* = 31), blue and red]. Importantly, the fixed-effects analysis included a separate predictor to estimate the variance explained by the number of saccades performed during the encoding phase (see Materials and Methods) based on individual trials. The differences in saccade-related activity between DS and ES conditions should therefore not account for the observed effects of attentional demand [Fig. 6B, with (green) and without (yellow) the saccade predictor], WM load and their interaction.

**Table 2:** Brain regions showing significant activation for saccades during encoding

Brain region	BA	*x*	*y*	*z*	*t*-value
R SFS	10	30	56	16	5.41
L FEF	6	−21	−7	51	5.42
R FEF	6	24	−7	52	6.87
L PrcS	6	−42	−10	46	6.64
R PrcS	6	45	−4	43	9.61
L SEF	6	−1	−1	49	6.56
R SEF	6	1	−1	49	6.22
L vlPrcS	6	−57	2	29	7.52
L IFG	9	−33	11	25	7.53
R Insula	13	45	14	4	5.54
L ant. IPS/IPL	40	−33	−37	43	7.99
R ant. IPS/IPL	40	36	−30	41	8.75
L middle IPS	7	−24	−55	46	6.48
R middle IPS	7	18	−49	43	10.16
R middle IPS	7	18	−52	55	9.66
R post. IPS	7	18	−61	37	9.98
L post. IPS/SPL	7	−21	−64	55	7.68
R post. IPS/SPL	7	21	−67	49	8.81
R ITG	19	48	−55	1	5.99
L Cuneus	18	−15	−98	10	7.83
R Cuneus	18	18	−91	10	8.75
L MOG	18	−15	−85	22	8.93
R MOG	18	12	−91	16	9.12
L lingual gyrus	18	−3	−79	−6	7.35
R lingual gyrus	18	15	−82	−3	8.01
R IOG	19	36	−76	−2	6.74
L FG	19	−27	−64	−5	8.29
L PHG	19	−28	−54	−5	7.61
L Thalamus		−12	−16	10	3.60
R Thalamus		9	−16	11	4.34
L LGN		−21	−22	−5	3.86
R LGN		−21	−22	−4	5.11
L Putamen		−24	5	7	5.36
R Putamen		24	5	4	4.87

Significant effects for the saccade predictor (contrast saccade predictor vs. baseline, *n *=* *8, fixed-effects analysis, *q*(FDR) < 0.05). Talairach coordinates (*x*, *y*, *z*; in mm) of the activation maxima and respective *t*-values are shown. BA, Brodmann area; FEF, frontal eye field; FG, fusiform gyrus; IFG, inferior frontal gyrus; IOG, inferior occipital gyrus; IPL, inferior parietal lobule; IPS, intraparietal sulcus; ITG, inferior temporal gyrus; LGN, lateral geniculate nucleus; MOG, middle occipital gyrus; PHG, parahippocampal gyrus; PrcS, precentral sulcus; SEF, supplementary eye fields; SFS, superior frontal sulcus; SPL, superior parietal lobule; vlPrcS, ventrolateral precentral sulcus.

## Discussion

The amount of information that can be held in visual WM is severely limited (Luck & Vogel, 1997; Cowan, 2001). Attention-based models of WM hold that this limited capacity is due to common capacity-limited resources shared with selective attention (Cowan, 2001; Rensink, 2002; Wheeler & Treisman, 2002). This view is supported by findings of functional interference observed in behavioural tasks that concurrently place demands on both processes (Smyth & Scholey, 1994; Awh & Jonides, 2001; Oh & Kim, 2004; Woodman & Luck, 2004) indicating common limited cognitive processes. Following this logic, we used fMRI to identify the common capacity-limited neural resources shared by spatial WM encoding and spatial attention. We combined visual search and delayed discrimination of spatial locations and manipulated orthogonally the demands on selective attention and WM encoding within one single task. This approach allowed us to test for shared neural substrates by means of overlapping activation for the two task components similar to previous studies (LaBar *et al.*, 1999; Pollman & von Cramon, 2000: Corbetta *et al.*, 2002; Lepsien *et al.*, 2005; Ikkai & Curtis, 2011; Soto *et al.*, 2008) and, in addition, by means of analysing interaction effects between the attention and WM manipulations. We hypothesized that if visual WM and selective attention were subserved in part by common areas with limited neural processing capacity, activation in these regions under conditions of joint demand on both processes should reach a plateau or at least be less than additive, as reflected in a statistical interaction between attention and WM. Conversely, we expected to find an additive increase in BOLD activation under simultaneous WM and attentional demands in regions whose processing capacity was not exceeded.

A significant interaction between spatial attention and encoding into spatial WM appeared in several visual, parietal and premotor regions and was reflected in an increase in BOLD activation across WM load conditions that was significantly smaller in the DS condition than the ES condition. Thus, BOLD activation reached a plateau or at least was less than additive under conditions of joint demand on both processes. This non-independence between search difficulty and WM load strongly indicates that the two cognitive domains indeed tap into common neural resources. Therefore, we propose that the interaction between the two task components, which occurred only when the demands on both processes were high, may reflect processing limits that stem from the competition for resources that are shared by the encoding into spatial WM and spatial attention in distributed posterior and premotor regions.

Importantly, the interaction effect did not appear in all regions that showed overlapping activation. In a subset of the overlap regions, mainly in the PFC, insula and subcortical regions, the BOLD signal increased to the same degree across WM load conditions in the ES and DS conditions. The additive increase in BOLD activation is in line with the assumption that the BOLD signal is a linear function of the items to be encoded (although this only holds true for the range within individual capacity) and the search difficulty, as has been previously shown in separate studies on attention (Culham *et al.*, 2001) and WM maintenance (Linden *et al.*, 2003; Todd & Marois, 2004; Xu & Chun, 2006). We suggest that the lack of an interaction between the two task components in a subset of the overlap regions demonstrates that activity in these regions did not reach a limit even in the conditions in which high WM load was combined with DS.

Given that WM performance and capacity estimates were equal in the ES and DS conditions it might be argued that high attentional demands did not impair the processes required during WM encoding, calling into question the interpretation in terms of common processing limitations. In the present study it was necessary to present the stimulus array for a fixed amount of time in order to ensure equal sensory stimulation across conditions. Thus, behavioural performance could be measured only when presenting the probe and, therefore, response accuracy and RTs captured only the final outcome of the task-related processes. Due to this methodological constraint it was not possible to validate directly encoding-related effects on brain activation by corroborative behavioural effects. However, in a previous behavioural study subjects were asked to indicate by button press when they had finished WM encoding (Mayer *et al.*, 2007b). In this study high attentional demands produced considerable costs in the time needed for successful WM encoding, but these costs did not simply reflect the time needed for visual search. The super-additive increase in the search time and the time needed for WM encoding in the conditions when WM load was combined with DS was taken as evidence for interference between attention and WM encoding (Mayer *et al.*, 2007a). Similar to the present findings, participants achieved equal WM performance at test in both search conditions. We therefore concluded that they engaged in a strategy that was needed to cope with the common processing limitations of attention and WM encoding. Given the sufficient time for successful WM encoding in the present fMRI study (5 s) we suggest that subjects also engaged in processes that allowed them to compensate for the common demands on limited neural resources shared by attention and WM processes in the posterior cortex.

Competition for processing resources between spatial WM encoding and attention seems to be the best explanation for these interaction effects, whereas haemodynamic saturation of the neurovascular system, insufficient time available for WM encoding in the DS condition, and limitations on perceptual rather than memory processes in the visual cortex do not seem to play a major role (for a detailed discussion of these points see Mayer *et al.*, 2007a). Moreover, findings of the eye movement experiment argued against the alternative explanation that activation associated with the demands on attention and WM load were mainly driven by oculomotor signals. Saccade frequency was not significantly affected by the number of locations subjects needed to encode whereas the number of eye movements was considerably higher in the condition of DS vs. ES. This difference was associated with increased activation in typical regions of oculomotor control (Corbetta *et al.*, 1998; Nobre *et al.*, 2000; Beauchamp *et al.*, 2001; Kimmig *et al.*, 2001; Ikkai & Curtis, 2008; Fairhall *et al.*, 2009). However, modelling the effect of saccade frequency on an individual trial basis to remove any variance in BOLD signal linearly associated with the number of eye movements, we still observed an increase in activation for DS vs. ES in these premotor and parietal regions. We therefore conclude that the observed activation was indeed related to attentional processing rather than the pure programming and execution of eye movements.

The idea that the interaction effect between WM and attention manipulations observed in our task reflects the competition for shared resources is also consistent with our previous report on common processing limitations of visual attention and the encoding of objects into WM (Mayer *et al.*, 2007a). In this study, activity in posterior parietal, visual and premotor regions showed a reduced WM load effect (WM load 3 vs. load 1) in the condition with high attentional demand. Because information load of object location and shape differ (Mayer *et al.*, 2007b) we increased WM load in the present study and found a corresponding interaction effect between WM load and search difficulty. Interestingly, the interaction effect between WM load and search difficulty was localised in similar regions both when participants needed to encode objects (Mayer *et al.*, 2007a) or locations (present study) indicating common capacity-limited resources for attention and WM encoding in the posterior cortex across WM domains. Although the visual stimulation was the same in both studies allowing for a qualitative comparison, activity associated with WM load also differed to some degree across experiments according to the different instructions. In the present experiment, load-related PFC activity extended less into parts of the left middle frontal gyrus and inferior frontal gyrus, a region that was especially pronounced in the object task (Mayer *et al.*, 2007b) and that has been associated with WM for nonspatial material such as objects, colors and faces (Manoach *et al.*, 2004; Mohr *et al.*, 2006; D’Esposito *et al.*, 1998y). These findings indicate that participants accomplished the present task by encoding and memorising the information about the spatial location rather than shape identity.

Posterior parietal and premotor regions play a crucial role in goal-directed visuospatial attention (Kanwisher & Wojciulik, 2000; Corbetta & Shulman, 2002; Pessoa *et al.*, 2003) and have been identified as key regions of the capacity limit of object-based WM maintenance (Linden *et al.*, 2003; Todd & Marois, 2004; Marois & Ivanoff, 2005; Xu & Chun, 2006; Mitchell & Cusack, 2008; Magen *et al.*, 2009). In the spatial domain, the roles of parietal and frontal and prefrontal cortex in the limitation of WM capacity are still much less clear. Leung *et al.* (2004) reported an inverted U-shaped response function for delay-related activity in parietal and also prefrontal regions when WM load was increased from 1 to 4 in a spatial delayed-response task. In contrast, previous work has shown that attention-based rehearsal, the common capacity-limited mechanism that is critical for successful WM maintenance, is accomplished by allocating attention via activity in the FEF and parietal cortex to extrastriate and parietal regions (Awh & Jonides, 2001; Jha, 2002; Postle *et al.*, 2004; Postle, 2006). The finding that the interaction between WM load and search difficulty appeared in distributed premotor and posterior but not prefrontal regions thus indicates that prefrontal and posterior regions may have different contributions to the limitations of the processes involved in WM. In contrast to previous studies, we focused on WM encoding and provide evidence that the processes supported by the lateral PFC were not limited by the attentional processes that constrained activity in posterior brain regions during this task phase. Visual information does not get automatically selected and encoded into WM but rather requires an active, time-consuming process that depends on the amount of information to be encoded (Jolicœur & Dell’Acqua, 1998; Woodman & Vogel, 2005). Therefore, in analogy to the attention-based rehearsal mechanism operating during the delay period, a rehearsal-like attention-based mechanism might work at encoding as well. At this point, we can only speculate about the function of such a process. For instance, repeated covert scanning of multiple locations might be necessary to verify the success of the encoding process and to eliminate irrelevant information if wrongly encoded (Nasr *et al.*, 2008). In addition, attentional mechanisms might determine the precision with which memory representations are formed (Bays & Husain, 2008). In that case, we would expect strong interactions between attention and WM encoding in posterior regions if the resolution of the memory representation was high. Conversely, such interaction should not occur if representations were formed with low precision. Future studies are needed to disentangle the attentional mechanisms supporting WM encoding.

One key finding of this study was that the PFC was not part of the activation pattern that reflected the common processing limitations of visual WM and attention. In the context of WM, PFC activation has been linked to a variety of control processes (Miller & Cohen, 2001; Koechlin *et al.*, 2003). For instance, higher levels of stimulus complexity demand greater strategic or organizational processing in order to facilitate WM performance (Glahn *et al.*, 2002; Bor *et al.*, 2003). In the present task, the formation of configural representations or chunks of information might have been especially demanding when subjects needed to encode five locations leading to stronger activation in the PFC in this condition.

According to the model of Curtis & D’Esposito (2003), top-down control from PFC occurs independent of the type of material that is actually stored in the posterior cortex. In support of this model, the left anterior middle frontal gyrus was involved in the encoding of both locations and objects into WM but not attention (Mayer *et al.*, 2007a). Thus, together with the previous results (Mayer *et al.*, 2007a), the present findings point to differential roles of prefrontal (e.g. stimulus-independent strategic processing) and premotor and posterior (e.g. capacity-limited attention-based mnemonic processing) regions during visual WM encoding.

In conclusion, the current study extends previous findings on the extensive interplay between attention and visual WM (Awh & Jonides, 2001; Jha, 2002; Postle *et al.*, 2004; Awh *et al.*, 2006; Lepsien & Nobre, 2007; Soto *et al.*, 2008; Ikkai & Curtis, 2011) by showing common capacity-limited neural mechanisms shared between spatial WM encoding and attention in premotor and posterior regions. The large consistency in posterior cortex activation associated with common limitations for attention and the encoding of objects (Mayer *et al.*, 2007a) or locations into WM suggests that the attention-based model of WM encoding may be valid across WM domains. We also provide evidence for a role of prefrontal cortex in forming stable representations of spatial patterns when attentional and memory demands are competing for more posterior neural resources.

## Abbreviations

BOLD: blood oxygen level-dependent
DS: difficult (visual) search
ES: easy (visual) search
FEF: frontal eye fields
IPS: intraparietal sulcus
ITI: intertrial interval
PFC: prefrontal cortex
PPC: posterior parietal cortex
PrcS: precentral sulcus
ROI: region of interest
SFS: superior frontal sulcus
WM: working memory

## Supporting Information

Fig. S1. Two-way ANOVA interaction plots for the main effects of WM load and attentional demand. WM load is shown on the *x*-axis, averaged BOLD responses for the time window between 6 and 10; s are shown on the *y*-axis, respectively. *F*-values of the interaction effect [*F*(Int)] are depicted for the corresponding brain regions. ES: easy search, DS: difficult search, FEF: frontal eye field, IPS: intraparietal sulcus, MOG: middle occipital gyrus, MTG: middle temporal gyrus, post: posterior, PrcS: precentral sulcus, SPL: superior parietal lobule.

Fig. S2. Averaged event-related time courses of the BOLD signal in the six experimental conditions from selected regions showing overlapping effects of attentional demand and WM load. Bars represent standard errors of the mean. Pre-SMA: pre-supplementary motor area, SMA: supplementary motor area.

Fig. S3. Group results (*n* = 8, GLM with additional saccade predictor, see materials and methods) for the encoding predictor (0-5 s). Statistical maps of the contrasts DS vs. ES (yellow), WM load 5 vs. 1 (blue), and the significant 2-way interaction of search difficulty x WM load (red) are shown. Activations are those exceeding a whole-brain false discovery rate threshold of *q*(FDR) < 0.05. GLM: general linear model, ES: easy search, DS: difficult search, CS: central sulcus, FEF: frontal eye field, IFS: inferior frontal sulcus, IPS: intraparietal sulcus, LS: lateral sulcus, MOG: middle occipital gyrus, OTS: occipito-temporal sulcus, PPC: posterior parietal cortex, pre-SMA: pre-supplementary motor area, SEF: supplementary eye fields, SFS: superior frontal sulcus.

Please note: As a service to our authors and readers, this journal provides supporting information supplied by the authors. Such materials are peer-reviewed and may be re-organized for online delivery, but are not copy-edited or typeset by Wiley-Blackwell. Technical support issues arising from supporting information (other than missing files) should be addressed to the authors.
