# Supplementary figures and images for: Common capacity-limited neural mechanisms of selective attention and spatial working memory encoding
Source: Eur J Neurosci. 2011 Sep;34(5):827–38. doi: 10.1111/j.1460-9568.2011.07794.x (PMC3465779; doi:10.1111/j.1460-9568.2011.07794.x)

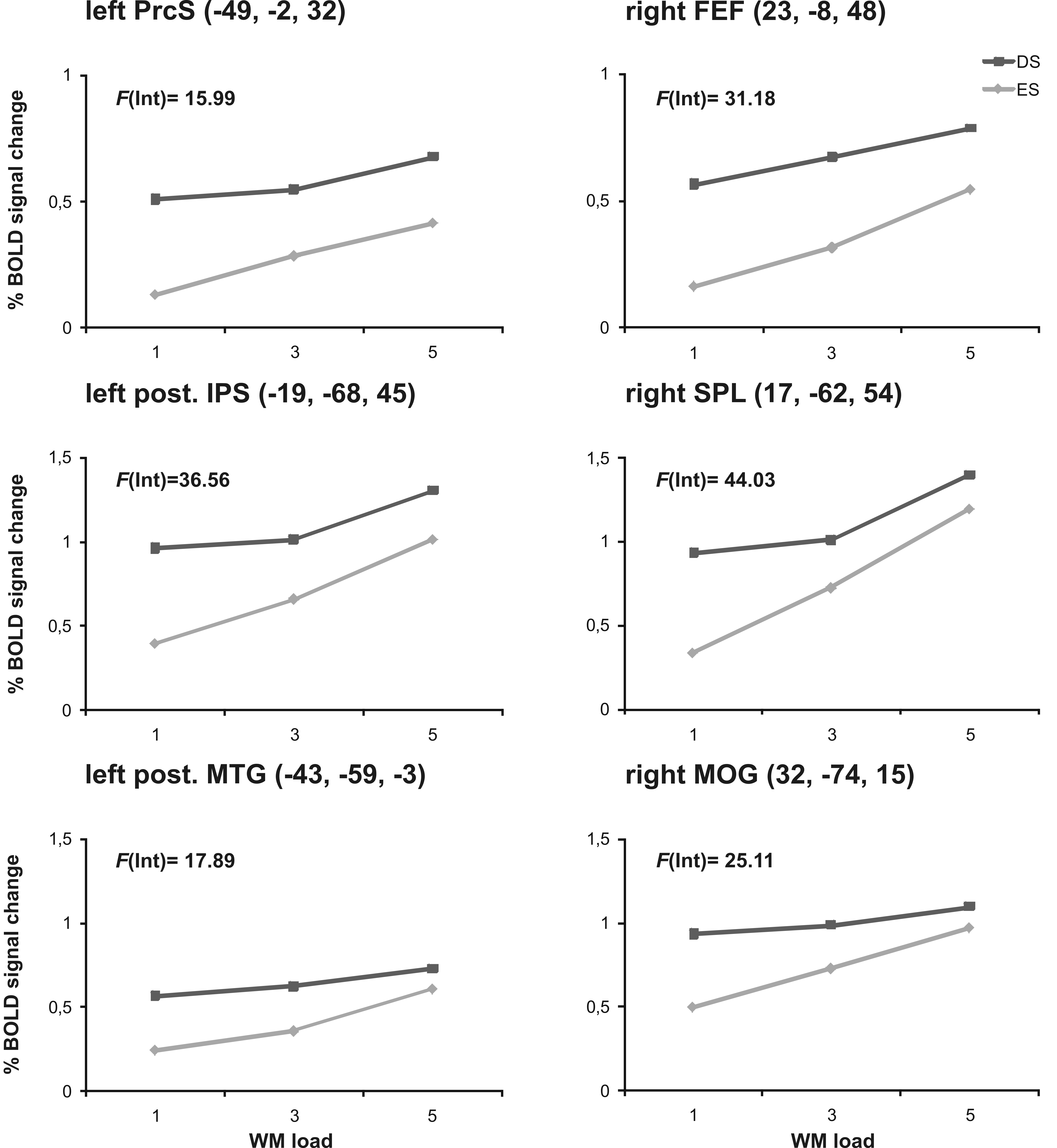

Supplement: Supplementary file 1 [file ejn0034-0827-SD1.tif]

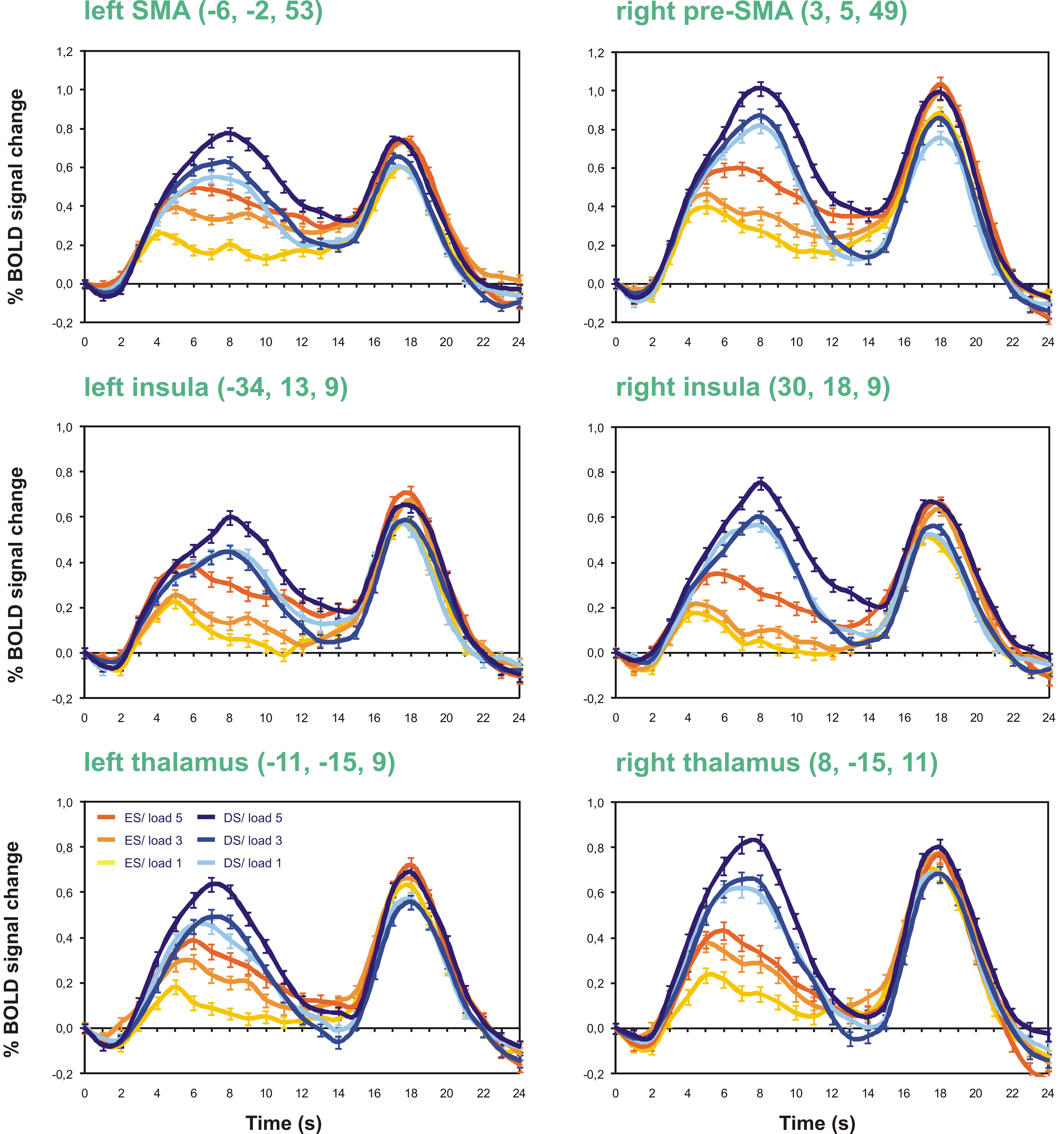

Supplement: Supplementary file 2 [file ejn0034-0827-SD2.tif]

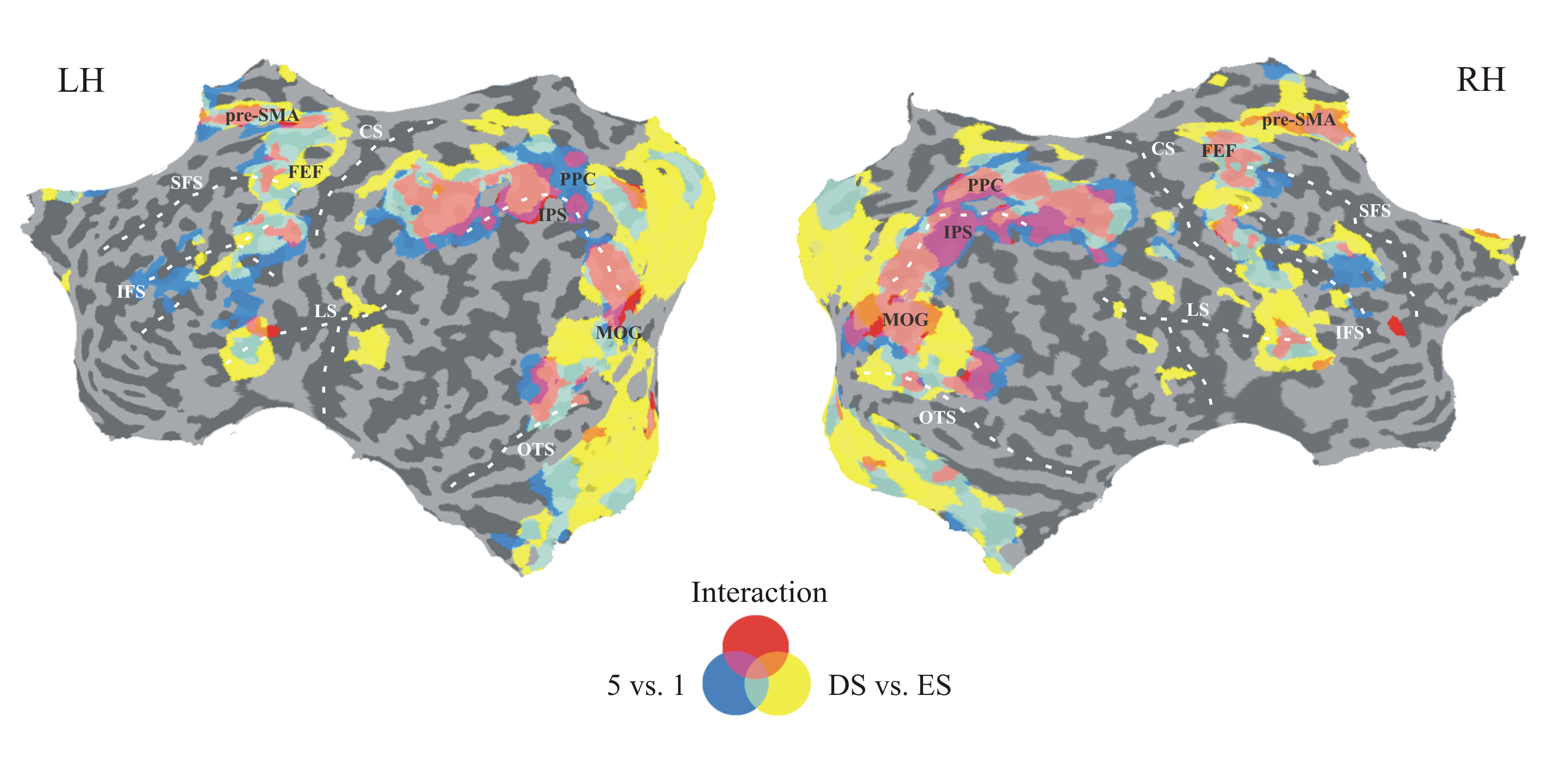

Supplement: Supplementary file 3 [file ejn0034-0827-SD3.tif]
